# Interrupted transport by the emergency medical service in stroke/transitory ischemic attack: A consequence of changed treatment routines in prehospital emergency care

**DOI:** 10.1002/brb3.1266

**Published:** 2019-04-13

**Authors:** Linda Alsholm, Christer Axelsson, Magnus Andersson Hagiwara, My Niva, Lisa Claesson, Johan Herlitz, Carl Magnusson, Lars Rosengren, Katarina Jood

**Affiliations:** ^1^ Department of Clinical Neuroscience Institute of Neuroscience and Physiology, The Sahlgrenska Academy at the University of Gothenburg Gothenburg Sweden; ^2^ Prehospen‐Centre of Prehosp Research, Faculty of Caring Science, Work Life and Social Welfare University of Borås Borås Sweden; ^3^ Department of Ambulance Care Jönköping County Hospital Jönköping Sweden; ^4^ Department of Ambulance Care Halland County Hospital Varberg Sweden; ^5^ Department of Molecular and Clinical Medicine University of Gothenburg and Sahlgrenska University Hospital Gothenburg Sweden

**Keywords:** EMS, stroke/TIA, transport

## Abstract

**Background:**

The discovery that not all patients who call for the emergency medical service (EMS) require transport to hospital has changed the structure of prehospital emergency care. Today, the EMS clinician at the scene already distinguishes patients with a time‐critical condition such as stroke/transitory ischemic attack (TIA) from patients without. This highlights the importance of the early identification of stroke/TIA.

**Aim:**

To describe patients with a final diagnosis of stroke/TIA whose transport to hospital was interrupted either due to a lack of suspicion of the disease by the EMS crew or due to refusal by the patient or a relative/friend.

**Methods:**

Data were obtained from a register in Gothenburg, covering patients hospitalised due to a final diagnosis of stroke/TIA. The inclusion criterion was that patients were assessed by the EMS but were not directly transported to hospital by the EMS.

**Results:**

Among all the patients who were assessed by the EMS nurse and subsequently diagnosed with stroke or TIA in 2015, the transport of 34 of 1,310 patients (2.6%) was interrupted. Twenty‐five of these patients, of whom 20 had a stroke and five had a TIA, are described in terms of initial symptoms and outcome. The majority had residual symptoms at discharge from hospital. Initial symptoms were vertigo/disturbed balance in 11 of 25 cases. Another three had symptoms perceived as a change in personality and three had a headache.

**Conclusion:**

From this pilot study, we hypothesise that a fraction of patients with stroke/TIA who call for the EMS have their direct transport to hospital interrupted due to a lack of suspicion of the disease by the EMS nurse at the scene. These patients appear to have more vague symptoms including vertigo and disturbed balance. Instruments to identify these patients at the scene are warranted.

## 1 INTRODUCTION

Stroke is one of the most severe manifestations of cardiovascular disease. Globally, it has been estimated that around 6.5 million people die from stroke each year (Feigin et al., 2015).

Furthermore, among the survivors, a substantial proportion will also have disabilities during a long period of follow‐up (Luengo‐Fernandez et al., 2013).

For patients with an acute stroke, the time until the start of treatment is critical. For patients in whom brain ischemia is the underlying aetiology, early revascularisation is the treatment alternative (Lees et al., 2010; Saver, 2006). For each minute that passes in the early chain of care of large‐vessel supratentorial ischemic stroke, 1.9 million nerve cells will die (Goyal et al., 2015).

For patients with a hemorrhage, in selected cases, acute interventions including surgery, the abrupt withdrawal of anticoagulants and/or the rapid lowering of blood pressure are available (Mendelow et al., 2013; Qureshi et al., 2016).

The use of the emergency medical service (EMS) has been shown to be associated with more rapid transport to hospital (Bae et al., 2010), thereby reducing the system delay, that is, the delay between the first medical contact and final diagnosis and treatment (Fassbender et al., 2013).

In order to enhance the early chain of care in stroke, it is important that the health‐care provider in the EMS system recognises the signs of stroke and then initiates a fast track, resulting in an early diagnosis and eventual early treatment. Previous experience indicates that, in western Sweden, at least two‐thirds of patients who use the EMS and who are subsequently diagnosed as having a stroke or transitory ischemic attack (TIA) are already recognised as having a stroke or TIA before arriving at hospital (Wireklint Sundstrom, Herlitz, Hansson, & Brink, 2015).

During the last few decades, the burden on the EMS has increased considerably in Sweden and in many other countries. An increasingly elderly population and the fact that more people dial 112 due to more or less acute conditions are important contributors to this finding. It has therefore been shown that a relatively large proportion of patients who dial 112 do not require EMS transport to the emergency department (ED) (Hjalte, Suserud, Herlitz, & Karlberg, 2007). For these patients, alternative care levels have been created in order to make both the EMS and the ED more available for “true emergencies”.

This change has, however, increased the demands imposed on the EMS clinician to distinguish patients with “time‐critical conditions” from those without. It has thus been shown that, among patients who are left at the scene without being transported to hospital, a significant proportion will seek hospital care within the next 72 hr (Magnusson, Kallenius, Knutsson, Herlitz, & Axelsson, 2016).

In a recent survey including the EMS in Gothenburg, 17% of the primary missions were allocated to the levels of care other than direct transport to hospital (Magnusson et al., 2018). In the same survey, 12% of the patients with a time‐critical condition in a retrospective evaluation were judged as being “potentially inappropriately assessed” in the prehospital setting. The most common final diagnoses in these cases were stroke and sepsis (Magnusson et al., 2018).

Since 2013, within the city of Gothenburg, we have registered all the patients who were hospitalised on a stroke ward due to stroke or TIA in a local register called Väststroke. We have found that about 70% of patients use the EMS (Väststroke, 2016).

The aims of this study were thus to:

1. Estimate the proportion of patients with a final diagnosis of stroke/TIA, whose ambulance transport to hospital was interrupted by the EMS nurse or by refusal by the patient or relatives/friend to be transported to hospital by the EMS
2. Describe 25 such cases with particular emphasis on the initial symptoms and final outcome.

## 2 METHODS

### 2.1 Setting

The study was conducted in a single‐site urban setting in the western part of Sweden. The EMS organisation covers 975 km^2^ with a population of 675,000 inhabitants. The population density is 733/km^2^. The median EMS mission time is 47 min. The EMS carries out about 80,000 assignments annually, of which 58,817 assignments were identified as primary assignments in 2015. Primary assignments mean that the EMS nurse meets and assesses a patient to an (hopefully) appropriate level of care. According to regulations, all ambulances in Sweden are manned by at least one registered nurse and 43% in the present organisation have postgraduate nurses specialising in prehospital emergency care and/or intensive care. The present EMS organisation has 22 units of which 18 are ambulances with advanced life saving capacity and four are special units. These four units include two single responders manned by one specialist trained registered nurse, one emergency‐physician manned unit and one scene‐commanding unit.

Within the studied urban area, two different prehospital pathways are available for patients with a suspicion of stroke:

1. Directly to brain CT if acute stroke symptoms within four hours (modified National Institute of Health Stroke Scale ((m)NIHSS) ≥ *2 points) or independent of delay if (m)NIHSS ≥ *6 points;
2. Directly to a stroke unit if recent appearance of paresis or aphasia, even if the symptoms have declined or disappeared if not fulfilling the criteria for the first alternative.

### 2.2 Different chains of prehospital care in EMS

There are two different principles:

1. Direct transport to hospital by ambulance;
2. Interrupted transport which means any of the following three:

(a) Delayed transport to hospital in another vehicle manned by an emergency medical technician in either a sitting or a horizontal position; (b) Referral to primary care either directly or with a delay; and (c) Patient remains at the scene with advice on self‐care or extended home care.

### 2.3 Patient selection

1. The denominator was all patients with a stroke/TIA who were included in Väststroke from January 1, 2015 to December 31, 2015 for whom an ambulance was requested. Among these cases, we looked for the proportion that had an interrupted transport to hospital according to a specified variable (interrupted transport; yes/no) within the register.
2. From 1 January 2013 to 31 December 2014, interrupted transports were recorded separately from two of the three hospitals which participated in Väststroke covering about half the catchment area. Until 2015, the information about interrupted transport (yes/no) was not included as a specific variable in the register.

The 25 unique cases which were reported in this study involved patients who were included in the register but for whom information on interrupted transport was collected from a separate report. Originally, 50 cases were collected, but, since information in the EMS records was insufficient regarding initial symptoms in half of them, only 25 cases remained.

### 2.4 Data collection

The patients who are described in this study were recruited from a quality register in western Sweden called Väststroke. This is a complementary register to the Swedish Stroke Register, which covers a large majority of patients in Sweden who are hospitalised with a final diagnosis of stroke/TIA.

The aim of Väststroke is to collect information about the chain of care in stroke/TIA, which is not collected in the Swedish Stroke Register. One example is the very early chain of care in stroke/TIA, that is, the prehospital phase. Information from the prehospital setting includes whether the patient was seen by the EMS, clinical findings on the arrival of the EMS, whether the EMS crew suspected a stroke/TIA, whether the nurse initiated a fast track to hospital and whether the patient was transported to hospital by the EMS.

Patients with interrupted transport were identified by a direct question relating to interrupted transport (yes/no), which was introduced into the register from 2015. These patients fulfilled any of the three criteria for interrupted transport as previously defined. A few of these patients (or relatives) did not wish to be transported by ambulance.

Among the 25 cases, specific questions were asked about initial symptoms and sequelae of the stroke at discharge from hospital. Information on different clinical variables in Väststroke was gathered from the electronic hospital and ambulance records.

### 2.5 Inclusion criteria

1. All EMS missions within the catchment area where the patient was finally hospitalised with a diagnosis of stroke/TIA
2. The patient was assessed by an EMS nurse. Either (a) the nurse did not assess the case as being in need of EMS transport directly to hospital and thus recommended any of the three alternatives for interrupted transport as previously defined or (b) the patient or relatives/friends did not want direct ambulance transport to hospital.

### 2.6 Exclusion criterion

Patients were excluded if there was limited information in the EMS record so that an interpretation of the situation at the time of the decision on the level of care was not possible.

### 2.7 Definition of (m)NIHSS

#### 2.7.1 Orientation

Ask about present month and the patient's age.

0 = Both are correct; 1 = One is correct; and 2 = None is correct.

### 2.8 Understanding

Give commands: close your eyes; clench your fist (non‐paretic side).

0 = Both are correct; 1 = One is correct; and 2 = None is correct.

### 2.9 Eye position and motion

Firstly, observe the position of the eyes and then their movement.

0 = Both are normal; 1 = The patient looks at the paretic side but can, on request, look at the other side; and 3 = The patient looks at the paretic side and cannot look at the other side.

#### 2.9.1 Visual field

Test one eye at a time. Use movement of fingers.

0 = Normal visual field; 1 = Limited visual field on one side; and 2 = Limited visual fields on both sides.

#### 2.9.2 Weakness in arm

The patient is lying. Straight elevation of arms 45 degrees. Ask the patient to hold the arms in that position for 10 s. Test the non‐paretic side first. Points refer to the worst side.

0 = Arms remain lifted for 10 s; 1 = Arms drop within five seconds but not all the way to the bed; 2 = Arms drop towards the bed within 10 s but with some resistance; 3 = Arms drop immediately but can be moved towards the bed; and 4 = No movement in the arm.

#### 2.9.3 Weakness in leg

The patient is lying. Straight elevation of legs 30 degrees. Ask the patient to hold the legs in that position for five seconds. Test the non‐paretic side first. Points refer to the worst side.

0 = Legs remain elevated for five seconds; 1 = Legs drop within five seconds but not all the way to the bed; 2 = Legs drop towards the bed but with some resistance; 3 = Legs drop immediately but can be moved towards the bed; and 4 = No movement in the leg.

#### 2.9.4 Sensitivity

Test with touch or with a blunt needle on the back of the hand or foot.

0 = Normal and 1 = Decreased sensitivity.

#### 2.9.5 Language communication

0 = Normal; 1 = Slight to moderate dysphasia. Difficulty finding words; 2 = Severe aphasia, only replies yes or no; and 3 = Total aphasia.

### 2.10 Determination of TIA/stroke

Patients with a clinical presentation which raised a suspicion of stroke/TIA were evaluated by a stroke physician and the diagnosis was made according to the WHO definition.

All patients underwent neuroimaging with brain computed tomography and/or brain magnetic resonance imaging.

### 2.11 Statistical methods

The results are expressed as numbers, percentages, and mean values.

### 2.12 Ethics

The study was approved by the Ethical Review Board in Gothenburg (Dnr 284‐17) on May 8, 2017.

Informed consent was not obtained from the patients.

## 3 RESULTS

In 2015, 34 of 1,310 EMS missions (2.6%) among cases who were subsequently hospitalised with a final diagnosis of stroke/TIA were interrupted without transporting the patient directly to hospital.

In most of these cases, the patients were transported to hospital by relatives or others a day or two later.

In all, 25 patients from half the catchment area were selected for a more detailed analysis. These 25 patients are listed in Table 1. As shown in the table, there were 14 women and 11 men. The age range was 51–98 years (the mean age was 77.4 years).

**Table 1:** Protocol. Compilation of results

Patient	Age	Sex	Symptom	Final diagnosis	Symptoms at discharge	Assessed according to NIHSS
1	67	Woman	Weakness in extremity	Stroke—infarction	Yes	Yes—0 points
2	85	Woman	Headache, abdominal discomfort	Stroke—hemorrhage	Yes	No
3	51	Man	Personality changes	Stroke—hemorrhage	Unclear	No
4	83	Woman	Personality changes, disturbed balance	Stroke—infarction	Yes	No
5	85	Woman	Disturbed balance, headache	TIA	Yes	No
6	70	Woman	Facial numbness	Stroke—infarction	No	Yes—0 points
7	88	Woman	Disturbed balance	Stroke—infarction	Yes	No
8	92	Man	Disturbed balance	Stroke—infarction	Yes	No
9	76	Woman	Vertigo	Stroke—infarction	Yes	Yes—0 points
10	71	Woman	Numbness in legs, numbness in cheek	TIA	No	No
11	65	Woman	Loss of vision	TIA	No	No
12	98	Man	Speech disturbances	Stroke—infarction	Yes	No
13	88	Man	Vertigo, disturbed balance	Stroke—infarction	Yes	Yes—0 points
14	80	Man	Arm weakness	Stroke—infarction	Yes	No
15	69	Woman	Vertigo, disturbed balance	Stroke—infarction	Yes	No
16	79	Man	Vertigo, disturbed balance	Stroke—infarction	Yes	Yes—0 points
17	66	Man	Personality changes, tiredness	Stroke—infarction	Yes	No
18	53	Woman	Facial nerve paresis	Stroke—infarction	Yes	Yes—0 points
19	78	Woman	Vertigo, disturbed balance	Stroke—infarction	Yes	Yes—0 points
20	76	Man	Vertigo, disturbed balance	Stroke—infarction	Yes	No
21	71	Man	Vertigo, disturbed balance, nausea	Stroke—infarction	Yes	No
22	92	Man	Facial nerve paresis	Stroke—infarction	Yes	Yes—0 points
23	86	Man	Arm weakness	Stroke—infarction	Yes	Yes—0 points
24	79	Woman	Aphasia, headache	TIA	No	No
25	88	Woman	Loss of vision	TIA	Yes	No

NIHSS: National Institute of Health Stroke Scale; TIA: transitory ischemic attack.

### 3.1 Initial symptoms

The most frequent dominant symptom was vertigo/disturbed balance (*n* = 11) (Tables 1 and 2). This was followed in order of frequency by headache (*n* = 3), a change of personality (*n* = 3), and hemiparesis (*n* = 3).

**Table 2:** Compilation of symptoms

Symptoms	Number
Vertigo and/or disturbed balance	11
Weakness in extremity	3
Headache	3
Personality changes	3
Numbness	2
Loss of vision	2
Speech disturbances	2
Facial nerve paresis	2
Nausea	2
Abdominal discomfort	1
Numbness in legs	1
Tiredness	1

The following symptoms dominated in a few patients: paresthesia (*n* = 2), loss of vision (*n* = 2), speech disturbances (*n* = 2), facial droop (*n* = 2), and nausea (*n* = 2).

### 3.2 Final diagnosis

The majority of patients had a final diagnosis of a cerebral infarction (*n* = 18). Two patients had a cerebral hemorrhage and five had a TIA.

### 3.3 Reasons for interrupted transport

In four of the cases, the patient or relatives declined transportation to hospital by the EMS.

In the remaining 21 cases, the reason (based on EMS notes) appeared to be a lack of suspicion of an underlying severe disease.

### 3.4 Neurological examination

In only nine cases were a neurological examination according to a local simplified version of the (m)NIHSS performed. Other details of the neurological examination were retrospectively assessed according to the documentation in the EMS records.

### 3.5 Symptoms at hospital discharge

Twenty of the 25 patients (80%) had major or minor sequelae from the stroke/TIA.

## 4 DISCUSSION

This is the first report from a pilot study in which we describe the occurrence of interrupted transport among patients who call for the EMS due to stroke/TIA. We found that, among patients with stroke/TIA who called for and were seen by the EMS health‐care providers, between 2% and 3% were in fact not directly transported to hospital. According to a subset analysis, in many of these cases, there was a lack of suspicion of an underlying severe disease and a number suffered a severe acute stroke with major sequelae.

### 4.1 Atypical symptoms of stroke

Our results provide an indication of the difficulties involved in the initial evaluation of patients with stroke. Among the 25 selected patients whose transport was interrupted, almost half (*n* = 11) suffered from problems with vertigo/disturbed balance. Another six patients suffered from either a change in their personality or a headache. As a result, the majority of patients suffered from symptoms that may not naturally raise a suspicion of stroke.

Acute symptoms of vertigo are not always easy to interpret. It has been reported that about three per cent of all adult patients seeking care at the emergency department do so due to vertigo (Newman‐Toker, Stanton, Hsieh, & Rothman, 2008). Among these patients, about three per cent have an underlying cerebrovascular aetiology (Kerber, Brown, Lisabeth, Smith, & Morgenstern, 2006). We found that, among all the patients who had a primary assignment by the EMS in Gothenburg in 2015, 3.4% had an emergency signs and symptoms (ESS) code indicating vertigo.

The proportion who had an ESS code indicating suspicion of a stroke/TIA or a neurological deficit was the same (3.4%). It has also been shown that, among patients who suffer from stroke and in whom the initial symptoms were vertigo, the risk of misdiagnosis is higher (Venkat et al., 2018) and about 30% of the cases were misinterpreted as having another disease (Saber Tehrani et al., 2013).

Vertigo as a sign of acute stroke has been reported to differ from vertigo caused by vestibular neuronitis by having a more acute onset in stroke (Tarnutzer, Berkowitz, Robinson, Hsieh, & Newman‐Toker, 2011). However, on both occasions, the symptom deteriorates when moving the head (Newman‐Toker et al., 2008) and the two conditions can be very difficult to distinguish without a detailed clinical examination such as the head impulse, nystagmus, test‐of‐skew test (Tarnutzer et al., 2011).

### 4.2 Do health‐care providers in the EMS recognise stroke?

Previous studies have shown that about 60%–70% of patients with stroke are recognised by the EMS crew (Brandler et al., 2015; Wireklint Sundstrom et al., 2015). In Sweden, a registered nurse on board each ambulance is responsible for the prehospital assessment and care. These nurses often have special education in prehospital care. However, in some cases, their experience of acute neurology may be limited. On the other hand, it was reported that even physicians in the ED did not recognise stroke in 18% of cases which were hospitalised and had a final diagnosis of stroke (Wireklint Sundstrom et al., 2015).

The observation that 2%–3% of all transports among patients with a final diagnosis of stroke/TIA were interrupted should therefore perhaps be regarded as a relatively low figure, particularly since symptoms of vertigo are fairly common in prehospital emergency care.

### 4.3 Which instruments did they use?

In the 25 cases, the simplified version of the NIHSS was used in nine. We do not know whether the criteria for using the NIHSS were fulfilled. Experience from Sweden indicates that there are difficulties involved in using the NIHSS appropriately in the prehospital setting (Hagiwara, Suserud, Jonsson, & Henricson, 2013). Ongoing research is evaluating the opportunity to involve a neurologist at the hospital to support the EMS crew via video communication (Söderholm et al., 2018). The most commonly used method in the prehospital setting in Sweden is the Face Arm Speech Test (FAST) (Yperzeele et al., 2014). We do not know the extent to which the FAST was used in the 25 cases.

### 4.4 Consequences of interrupted EMS transport

It is obvious that interrupted transport to hospital by the EMS resulted in a delay to diagnosis and a delay to eventual treatment. The way in which this delay influenced the final extent of the cerebral damage and the clinical outcome can only be speculated upon.

However, the consequences from a caring science perspective may be equally important. Subsequent telephone contact with these patients indicated that some expressed disappointment, as they felt that their symptoms were not initially appropriately assessed. Furthermore, some patients wondered whether the sequelae of the stroke could perhaps have been prevented to some extent, if the disease had been appropriately assessed from the beginning, and this will most probably cause unpleasant feelings.

### 4.5 Do we underestimate the frequency of misdiagnosis of stroke/TIA in the prehospital setting?

This article presents cases with stroke/TIA who dialled 112 and were assessed by the EMS nurse as not being ill enough to require direct transport to hospital by the EMS but were subsequently transported to hospital later by other means and were then hospitalised. One question that arises is whether there are other patients with stroke/TIA whose transport is interrupted but who never seek further health care. Furthermore, there are patients who dial 112 for whom an ambulance is never dispatched or dispatched with the wrong dispatch code (Berglund, von Euler, Schenck‐Gustafsson, Castren, & Bohm, 2015). Finally, there may be patients whose transport is interrupted but who dial 112 a day or two later and are then appropriately assessed and transported to hospital. In these cases, the initial inappropriate assessment not to convey these patients may never be discovered. As a result, there are a number of reasons for assuming that we are underreporting the true number of patients with stroke/TIA who are inappropriately assessed by health‐care providers at the first contact with health care after having called for the EMS.

### 4.6 Strengths and limitations

To the best of our knowledge, this is the first time that problems with interrupted EMS transport among patients with a final diagnosis of TIA/ stroke have been reported.

However, in this pilot study, only a subsample of the patients who fulfilled the criteria for inclusion were evaluated in terms of initial symptoms and final outcome. We do not know whether they are representative of the total study cohort. The sample size is limited. The setting for the study was an urban area. We do not know whether the routines in prehospital care would be the same in a rural area and only part of Sweden was included in the analysis. Previous experience indicates that the population who call for the EMS differ in different geographical regions in Sweden (Beillon, Suserud, Karlberg, & Herlitz, 2009).

One important finding was that a large group of the patients who fulfilled the inclusion criteria had to be excluded due to limited information about their initial symptoms.

The time of onset of symptoms, the patients’ comorbidity and the patients’ functional status before the onset of symptoms were not reported and all these factors may have influenced the decision about the level of care.

Finally, one of the patients with a TIA had residual symptoms, which may indicate an error in the final diagnosis.

It is possible to argue about whether the prehospital arena is the best place to perform the kind of evaluation that was performed in this study. However, this is where the chain of care begins in time‐critical conditions such as stroke and the decisions that are made here will often have a great impact on the subsequent treatment and outcome.

### 4.7 Clinical implications

If our experience is extrapolated to the whole country, it can be assumed that, in Sweden (10 million inhabitants), a minimum of 500 patients (most probably even more) with stroke/TIA each year call for the EMS but are not transported directly to hospital due to an inappropriate assessment at the scene. In a large number of these cases, this is explained by atypical symptoms, of which vertigo may be the most frequent. We need to know more about how to identify patients with symptoms of vertigo which are caused by stroke/TIA. We thus need to introduce better screening instruments in prehospital emergency care to evaluate patients with acute vertigo in a more optimal fashion.

## 5 CONCLUSION

Based on data from this pilot study, we hypothesize that a fraction of patients with stroke/TIA who call for EMS have their transport interrupted due to a lack of suspicion of the disease by the EMS clinician on the scene. These patients appear to have more vague symptoms including vertigo and disturbed balance. Instruments to identify these patients are warranted.

## CONFLICT OF INTEREST

The authors hereby certify that we have all seen and approved this manuscript. We guarantee that the paper is the authors’ original work and that it has not been the subject of prior publication and is not under consideration for publication elsewhere. On behalf of all the co‐authors, the corresponding author bears full responsibility for the submission. There are no financial or other relationships that might pose a conflict of interest.
